# Life course effects of genetic susceptibility to higher body size on body fat and lean mass: prospective cohort study

**DOI:** 10.1093/ije/dyad029

**Published:** 2023-03-23

**Authors:** Scott Waterfield, Tom G Richardson, George Davey Smith, Linda M O’Keeffe, Joshua A Bell

**Affiliations:** MRC Integrative Epidemiology Unit, University of Bristol, Bristol, UK; Population Health Sciences, Bristol Medical School, University of Bristol, Bristol, UK; Cancer Research UK Integrative Cancer Epidemiology Programme, University of Bristol, Bristol, UK; MRC Integrative Epidemiology Unit, University of Bristol, Bristol, UK; Population Health Sciences, Bristol Medical School, University of Bristol, Bristol, UK; MRC Integrative Epidemiology Unit, University of Bristol, Bristol, UK; Population Health Sciences, Bristol Medical School, University of Bristol, Bristol, UK; MRC Integrative Epidemiology Unit, University of Bristol, Bristol, UK; Population Health Sciences, Bristol Medical School, University of Bristol, Bristol, UK; School of Public Health, University College Cork, Cork, Ireland; MRC Integrative Epidemiology Unit, University of Bristol, Bristol, UK; Population Health Sciences, Bristol Medical School, University of Bristol, Bristol, UK

**Keywords:** Body size, adiposity, genetics, DXA, GRS, ALSPAC

## Abstract

**Background/objectives:**

Different genetic variants are associated with larger body size in childhood vs adulthood. Whether and when these variants predominantly influence adiposity are unknown. We examined how genetic variants influence total body fat and total lean mass trajectories.

**Methods:**

Data were from the Avon Longitudinal Study of Parents and Children birth cohort (*N* = 6926). Sex-specific genetic risk scores (GRS) for childhood and adulthood body size were generated, and dual-energy X-ray absorptiometry scans measured body fat and lean mass six times between the ages of 9 and 25 years. Multilevel linear spline models examined associations of GRS with fat and lean mass trajectories.

**Results:**

In males, the sex-specific childhood and adulthood GRS were associated with similar differences in fat mass from 9 to 18 years; 8.3% [95% confidence interval (CI) 5.1, 11.6] and 7.5% (95% CI 4.3, 10.8) higher fat mass at 18 years per standard deviation (SD) higher childhood and adulthood GRS, respectively. In males, the sex-combined childhood GRS had stronger effects at ages 9 to 15 than the sex-combined adulthood GRS. In females, associations for the sex-specific childhood GRS were almost 2-fold stronger than the adulthood GRS from 9 to 18 years: 10.5% (95% CI 8.5, 12.4) higher fat mass at 9 years per SD higher childhood GRS compared with 5.1% (95% CI 3.2, 6.9) per-SD higher adulthood GRS. In females, the sex-combined GRS had similar effects, with slightly larger effect estimates. Lean mass effect sizes were much smaller.

**Conclusions:**

Genetic variants for body size are more strongly associated with adiposity than with lean mass. Sex-combined childhood variants are more strongly associated with increased adiposity until early adulthood. This may inform future studies that use genetics to investigate the causes and impact of adiposity at different life stages.

Key Messages

- Based on repeated body scanning from childhood to young adulthood, genetic predisposition to larger body size is primarily associated with higher fat mass, not higher lean mass.
- Childhood body size variants are most strongly associated with fat mass from around age 9–13 years, whereas adulthood body size variants become most strongly associated with fat mass by age 25 years.
- These findings help to validate studies which examine the impact of life course body size on disease.

## Introduction

Excess adiposity is now a global pandemic, with 64% of UK adults being overweight or obese, and 34% of UK children being overweight or obese by age 10 years.^1^ Overweight and obesity are associated with negative health outcomes, such as accelerated biological aging (as measured by epigenetic age analysis),^2^^,^^3^ and a number of life-limiting diseases such as cardiovascular disease and cancer.^4–7^ A host of environmental factors, such as socioeconomic disadvantage,^8^ energy-dense diets and physical inactivity^9^ likely influence the population rates of excessive adiposity.

Genome-wide association studies (GWAS) have established that individual susceptibility to higher adiposity is influenced in part by genetic variation, with hundreds of single nucleotide polymorphisms (SNPs) now found to be robustly associated with adiposity as measured indirectly using body mass index (BMI).^10^ Most of these genetic studies have focused on BMI measured in adulthood, the largest of which are meta-analyses of data from the Genetic Investigation of Anthropometric Traits (GIANT) consortium and the UK Biobank.^11^ Genetic variants associated with BMI measured in childhood were first identified by the Early Growth Genetics (EGG) consortium,^12^ and have recently been expanded upon using self-recalled childhood body size relative to peers from the much larger UK Biobank dataset. These latter findings suggest that large numbers of different SNPs are associated with recalled childhood body size, which have already been validated against BMI measures^13^^,^^14^ and which have only moderate overlap with adulthood body size SNPs (Genetic Correlation: rG = 0.61).^15^

These childhood body size SNPs, which are more numerous than sets previously available owing to the much larger sample size on which they are based, are being used for new life course Mendelian randomization (MR) studies as instruments for assumed childhood adiposity.^15^^,^^16^ However, it is not clear to what extent they are actually associated with objective measures of childhood adiposity, due to the non-objective phenotype measure used to discover them (self-recalled body size relative to peers at age 10 years). Furthermore, due to the lack of objective measures of body composition in the aforementioned studies, it is also unclear whether these SNPs predominantly raise adiposity (fat mass) as opposed to lean mass. Most studies have also not had repeat assessments of objective body composition measures such as fat and lean mass available across different life stages and thus, whether the influence of different body size SNPs is truly confined to specific periods of the life course is not well understood. Examining associations of body size SNPs with objective measures of body composition, taken repeatedly across different life stages, would enable insight into the time-sensitive effects of these SNPs on fat or lean mass, and aid the interpretation of new life course MR studies which use these SNPs as instruments to study the impact of life-stage adiposity on health outcomes.

In this study, we examined the associations of SNPs for childhood and adulthood body size using genetic risk scores (GRSs) with trajectories of dual-energy X-ray absorptiometry (DXA) total, trunk and peripheral (arms + legs) fat mass, and total lean mass, from ages 9 to 25 years (y) in the Avon Longitudinal Study of Parents and Children (ALSPAC) birth cohort. Analyses were performed separately by sex using sex-specific GRSs to examine how genetic influences on fat and lean mass at different life stages may differ between males and females.

## Methods

### Study participants

ALSPAC is a prospective birth cohort study in south-west England. Pregnant women resident in one of the three Bristol-based health districts, with an expected delivery date between 1 April 1991 and 31 December 1992, were invited to participate. The study has been described elsewhere in detail and ethical approval for the study was obtained from the ALSPAC Ethics and Law Committee and the local research ethics committees.^17–19^ ALSPAC initially enrolled a cohort of 14 541 pregnancies, from which 14 062 live births occurred, with 13 998 alive at 1 year. Follow-up has included parent- and child-completed questionnaires, links to routine data and clinic attendance. The present analyses were restricted to offspring participants who were firstborn and who were identified as being of a White ethnicity based on parental report via questionnaire, given that the source GWAS for body size was based on individuals of European genetic ancestry.

Research clinics were held when these offspring participants were approximately 9, 10, 11, 13, 15, 18 and 25 years old. Data for 25 years of age were collected and managed using REDCap electronic data capture tools hosted at the University of Bristol. REDCap (Research Electronic Data Capture) is a secure, web-based, software platform designed to support data capture for research studies.^20^ The study website contains details of all the data that are available through a fully searchable data dictionary [http://www.bristol.ac.uk/alspac/researchers/access/].

### Childhood and adulthood body size GRSs

Genotype was measured with the Illumina HumanHap550 quad chip platform. Genotype data were imputed using the Haplotype Reference Consortium (HRC) panel. Separate sets of SNPs for childhood body size and adulthood body size were used for males and females based on sex-specific UK Biobank GWAS (repeat ref.); 138 (131 present in ALSPAC) childhood female, 215 (187 present) adulthood female, 68 (60 present) childhood male and 159 (133 present) adulthood male SNPs. For comparison and completeness, a set of 298 (279 present) SNPs for childhood body size based on sexes combined, and a set of 557 (477 present) SNPs for adulthood body size based on sexes combined, were also examined. All SNPs are listed in Supplementary File 1 (available as Supplementary data at *IJE* online).^15^ The ‘adulthood’ SNPs were identified via a GWAS of measured BMI, and the ‘childhood’ SNPs were identified via a GWAS of the same individuals who were asked the question: ‘When you were 10 years old, compared with average would you describe yourself as thinner, plumper or about average?’ These scores were validated against BMI in external datasets and via simulations as described in Richardson *et al.*^15^ GRSs were constructed using PLINK 1.9, with effect alleles and beta coefficients from the GWAS used as external weightings. Standard scoring was applied by multiplying the effect allele count (or probabilities if imputed) at each SNP (values 0, 1 or 2) by its weighting, summing these, and dividing by the total number of SNPs used. Each score therefore reflects the average per-SNP effect on increasing a category of childhood or adulthood body size (separately). For single SNP analyses, the effect allele was chosen to have a positive effect (based on the beta value of the Richardson GWAS results).

### Body fat and lean mass measures

Total body fat (less head), central (trunk) fat, peripheral (legs + arms) fat and total lean mass (less head), each in kilograms, were measured using whole-body DXA scans performed at the ALSPAC clinics and undertaken at ages 9, 11, 13, 15, 18 and 25 years using a Lunar prodigy narrow fan beam densitometer. Scans were screened for anomalies, motion and material artefacts and realigned when necessary.

### Associations of childhood and adulthood body size GRSs with trajectories of body fat and lean mass

In males and females separately, we used multilevel linear spline models to examine associations of a standard deviation (SD) higher childhood and adulthood body size GRS with trajectories of DXA total, trunk and peripheral fat mass and total lean mass, from 9  to 25 years. As our goal was to examine independent associations of the childhood and adulthood GRS with changes over time in body composition, all analyses were performed with mutual adjustment for GRSs, i.e. the childhood GRS model was adjusted for the adulthood GRS and vice versa. Multilevel models estimate the mean trajectory of the outcome while accounting for the non-independence (i.e. clustering) of repeated measurements within individuals, change in scale and variance of measures over time, and differences in the number and timing of measurements between individuals [using all available data from all eligible participants under a missing at random (MAR) assumption].^21^ Linear splines allow knot points to be fit at different ages to derive periods in which change is approximately linear. Knot points were fitted at 13 and 15 as per previous work,^22^^,^^23^ with an additional knot point added at 18 for the extension of trajectories to 25 years. These linear spline multilevel models included two levels: measurement occasion and individual. All DXA models included sex- and age-specific adjustments for height as previously described.^24^ Inclusion criteria for analyses were as follows: availability of sex-specific GRS data for both childhood and adulthood, and at least one DXA measure for the specific outcome being analysed.

To aid interpretation, GRSs were internally standardized by centring around the sample mean and dividing by the SD: [individual GRS minus mean(GRS) divided by SD (GRS)]. Fat-based DXA measures had skewed distributions on most occasions and were therefore log-transformed before modelling took place. To aid comparability with fat-based measures, lean mass was also log-transformed. The sex-specific effect of each GRS (per SD) on each outcome trajectory was then estimated by including an interaction term between the standardized GRS and the intercept (value of the outcome at 9 years) and each linear spline period, providing estimates for the association between an SD increase in each GRS and the intercept and each linear spline period Following analysis, these estimates were then used to calculate the mean predicted trajectory of fat and lean mass for individuals with an SD higher (relative to the mean) childhood and adulthood GRS. Note that for outcomes which were log-transformed, these graphs are displayed in original units by back-transforming from the log scale. The difference in fat and lean mass at different ages from 9 to 25 years per SD higher childhood and adulthood GRS was also calculated. Differences and confidence intervals were calculated on the log scale, then back-transformed to a ratio of geometric means and converted to and displayed as percentage differences in predicted fat and lean mass at different ages from 9 to 25 years per SD higher childhood and adulthood GRS. Sensitivity analysis models as described above were also run using: (i) sex-specific GRS without mutual adjustment; (ii) non-sex specific GRS (among sexes separately and sexes combined); and (iii) non sex-specific GRS without mutual adjustment. All models are outlined formulaically in Supplementary File 2 (available as Supplementary data at *IJE* online). Single SNPs analyses using sex-specific and sex-combined GRS SNPs (replacing the GRS of interest with individual SNPs, expressed as per-allele effects) were also carried out. Last, we used linear models (of form: Outcome ∼ GRS) to determine the percentage of variance that each GRS explains in total fat mass and total lean mass as measured by R^2^ value. All trajectories were modelled in MLwiN (V3.05),^25^ called from R statistical software using the R2MLwiN library.^26^

This study is reported in line with STROBE guidelines (see Supplementary File 3, available as Supplementary data at *IJE* online). All code used for analyses is available on [github.com/SBWaterfield].

## Results

### Sample characteristics

A total of 6926 individuals (3511 females and 3415 males) was included in our analyses. Table 1 describes several sociodemographic characteristics of participants included in our analyses. Males and females included in analyses did not differ based on their parental socioeconomic backgrounds. Females had lower birthweight, younger ages at onset of puberty and higher fat mass at ages 9  and 25 y than males. Males had higher lean mass at 9 and 25 y than females. Supplementary Table S1 (available as Supplementary data at *IJE* online) compares characteristics of participants included in analyses vs those excluded from analyses due to missing data (either not having genetic data and/or at least one DXA measure). Participants included in analyses had parents who had higher educational attainment and household social class, and mothers who were less likely to have smoked during pregnancy than those who were excluded from analyses.

**Table 1:** Characteristics of Avon Longitudinal Study of Parents and Children (ALSPAC) participants included in analyses

	Females	Males
	*N* = 3511	*N* = 3415
	*n* (%)	*n* (%)
**Household social class** ^a^		
Professional	466 (15.1)	476 (15.9)
Managerial & Technical	1395 (45.3)	1332 (44.4)
Non-Manual	748 (24.3)	745 (24.8)
Manual	321 (10.4)	326 (10.9)
Part skilled & unskilled	148 (4.8)	121 (4.0)
**Mother’s highest educational qualification** ^b^		
Less than O level	706 (22.0)	729 (23.1)
O level	1148 (35.8)	1121 (35.6)
A level	828 (25.9)	807 (25.6)
Degree or above	521 (16.3)	494 (15.7)
**Mother’s partner’s highest educational qualification** ^b^		
Less than O level	931 (29.7)	831 (27.2)
O level	675 (21.5)	683 (22.3)
A level	872 (27.8)	855 (27.9)
Degree or above	658 (20.9)	690 (22.6)
**Maternal smoking during pregnancy (first 3 months)** ^c^	606 (18.6)	623 (19.5)
	**Mean (SD)**	**Mean (SD)**
**Birthweight (kg)**	3.39 (0.48)	3.51 (0.54)
**Puberty timing**: age at peak height velocity (years)^d^	11.75 (0.82)	13.58 (0.92)
**Body composition measures**		
DXA total fat mass age 9 clinic (kg)	9.7 (5.0)	7.3 (4.8)
DXA total fat mass age 25 clinic (kg)	24.8 (10.1)	20.7 (9.7)
DXA trunk fat mass age 9 clinic (kg)	3.9 (2.5)	2.9 (2.3)
DXA trunk fat mass age 25 clinic (kg)	11.5 (6.1)	10.6 (5.9)
DXA peripheral fat mass age 9 clinic (kg)	5.2 (2.4)	3.9 (2.4)
DXA peripheral fat mass age 25 clinic (kg)	12.4 (4.9)	9.2 (3.9)
DXA total lean mass age 9 clinic (kg)	23.6 (3.2)	25.5 (3.0)
DXA total lean mass age 25 clinic (kg)	41.2 (5.3)	57.1 (7.5)

The total *N* for these characteristics differs somewhat from sample sizes used in analyses as these complete data on these characteristics were not required for our analyses.

DXA, dual-energy X-ray absorptiometry.

a Household social class was measured as the highest of the mother’s or her partner’s occupational social class using data on job title and details of occupation collected about the mother and her partner from the mother’s questionnaire at 32 weeks of gestation. Social class was derived using the standard occupational classification (SOC) codes developed by the United Kingdom Office of Population Census and Surveys and classified as I-professional, II-managerial and technical, IIINM-non-manual, IIIM-manual, and IV&V part-skilled occupations and unskilled occupations.

b Education status was recorded via a questionnaire of the mother, asking for her highest level of education and her partner's highest level of education at 32 weeks of gestation. Responses are categorized into less than O level (including vocational courses and the certificate of secondary education (CSE), O level (taken at 16 years), A levels (taken at 18 years) or university degree (or higher).

c Maternal smoking in the first trimester was self-reported via questionnaire of the mothers; form of smoking was defined as: cigarettes, cigars, pipe, or ‘other’, all of which we combine into a binarized value of having smoked or not having smoked during pregnancy.

d Puberty timing was estimated via age at peak height velocity based on the Superimposition by Translation And Rotation (SITAR) methodology.^27^

### Estimated effects of childhood and adulthood body size GRSs on trajectories of fat mass

Supplementary Figure S1A and B (available as Supplementary data at *IJE* online) shows the mean sex-specific trajectories of total fat mass from 9–25 y for: (i) participants with the mean childhood GRS and mean adulthood GRS; (ii) participants with a 1-SD higher childhood GRS compared with the mean; and (iii) participants with a 1-SD higher adulthood GRS compared with the mean.

**Figure 1:**
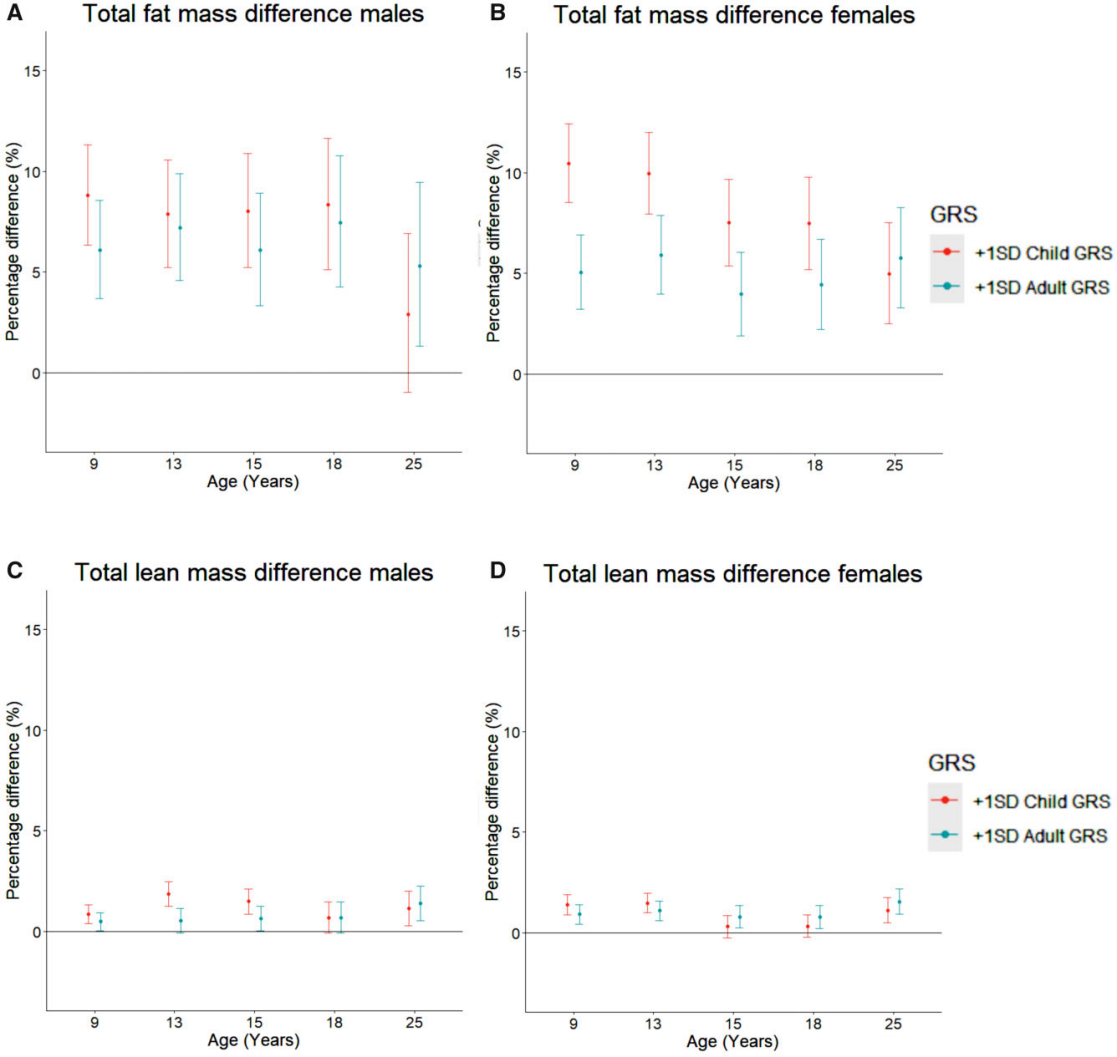
Mean difference in fat (A, B) and lean mass (C, D) in females and males from 9 to 25 years old by child and adult GRS. Differences shown are for a participant with a one standard deviation (1-SD) higher childhood genetic risk score (GRS) and a 1-SD higher adulthood GRS compared with the mean (participant with the mean childhood and adulthood GRS). Differences are derived from models which include mutual adjustment for each score. Mean trajectory is centred on the sex-specific mean of the child [male mean: 0.0098 (SD: 0.0008), female mean: 0.0086 (SD: 0.0005)] and adult [male mean: 0.0076 (SD: 0.0004), female mean: 0.0079 (SD: 0.0004)] GRS. The difference in fat mass per SD higher child or adult GRS is back-transformed from the log scale for ease of interpretation and is a ratio of geometric means, expressed as a percentage difference. GRS, genetic risk score

Compared with a male with the mean childhood and adulthood GRS, a 1-SD higher childhood GRS or adult GRS was associated with 8.8% [95% confidence interval (CI) 6.3, 11.3] and 6.1% (95% CI 3.7, 8.6) higher total fat mass at 9 y, respectively, and similar rates of change in total fat mass from 9 to 25 y among males (Figure 1A and Table 2). This resulted in the persistence of associations of childhood and adulthood GRSs with total fat mass at 9 y up to age 18 y; associations for both GRSs were of comparable magnitude at each age and were broadly similar across these ages. For example, associations of a 1-SD higher childhood and adulthood GRS with total fat mass were similar at 18 y, with 8.3% (95% CI 5.1, 11.6) and 7.5% (95% CI 4.3, 10.8) higher total fat mass at 18 y per SD higher childhood and adulthood GRS, respectively. At age 25 y, associations for the childhood GRS attenuated whereas associations for adulthood GRS persisted [difference per SD higher childhood GRS: 2.9% (95% CI -1.0, 6.9), difference per SD higher adulthood GRS: 5.3% (95% CI 1.3, 9.5)].

**Table 2:** Mean trajectory and mean difference in trajectory of total fat mass from 9 to 25 years per SD higher child GRS and adult GRS in males, with mutual adjustment for each score

Age (years)	Mean trajectory and percentage change (per year) in fat mass at age specified (95% CI)	Mean difference (95% CI) per SD higher child GRS	Mean difference (95% CI) per SD higher adult GRS
9	5.6 (5.4, 5.8) kg	8.8 (6.3, 11.3) %	6.1 (3.7, 8.6) %
9–13	15.3 (14.2, 16.4) %/y	−0.2 (-0.8, 0.3) %/y	0.3 (-0.3, 0.8) %/y
13	9.9 (9.7, 10.2) kg	7.9 (5.2,10.6) %	7.2 (4.6,9.9) %
13–15	−6.9 (-7.8, -5.9) %/y	0.1 (-0.9, 1.1) %/y	−0.5 (-1.5, 0.5) %/y
15	8.6 (8.4, 8.8) kg	8.0 (5.2,10.9) %	6.1 (3.3,8.9) %
15–18	10.6 (9.8, 11.4) %/y	0.1 (-0.7, 0.9) %/y	0.4 (-0.4, 1.2) %/y
18	11.6 (11.3, 12.0) kg	8.3 (5.1,11.6) %	7.5 (4.3,10.8) %
18–25	7.9 (7.4, 8.3) %/y	−0.7 (-1.2, -0.3) %/y	−0.3 (-0.7, 0.2) %/y
25	19.8 (19.1, 20.5) kg	2.9 (-1.0,6.9) %	5.3 (1.3,9.5) %

Mean trajectory is centred on the sex-specific mean of the child (male mean: 0.0098 (SD: 0.0008), and adult (male mean: 0.0076 (SD: 0.0004) GRS. The difference in fat mass per SD higher child or adult GRS is back-transformed from the log scale for ease of interpretation and is a ratio of geometric means, expressed as a percentage difference. GRS, genetic risk score; SD, standard deviation; CI, confidence interval; y, years.

Compared with a female with mean childhood and adulthood GRS, a 1-SD higher childhood GRS or adulthood GRS was associated with 10.5% (95% CI 8.6, 12.4) and 5.1% (95% CI 3.2, 6.9) higher total fat mass at 9 y, respectively (Table 3 and Figure 1B). Results for the association of each GRS with rates of change in total fat mass in females were broadly similar to those for males, demonstrating no strong associations of either GRS with change in total fat mass across most spline periods, except for associations with change in total fat mass from 13 to 15 y; a 1-SD higher childhood and adulthood GRS was associated with -1.1% (95% CI -1.9, -0.3) and -0.9% (95% CI -1.7, -0.2) per year slower rates of increase in total fat mass, respectively, from 13 to 15 y. This resulted in a slight reduction in the childhood and adulthood GRS associations with total fat mass at 15 and 18 y, though the childhood GRS associations remained stronger than the adulthood GRS associations at both ages at 7.5% (95% CI 5.2, 9.8) and 4.4% (95% CI 2.2, 6.7) higher total fat mass at 18 y per SD higher childhood and adulthood GRS, respectively. At age 25 y, the childhood and adulthood GRS were similarly associated with total fat mass in females, driven by slightly slower gains per year in total fat mass from 18 to 25 y per SD higher childhood GRS.

**Table 3:** Mean trajectory and mean difference in trajectory of total fat mass from 9 to 25 years per SD higher child GRS and adult GRS in females, with mutual adjustment for each score

Age (years)	Mean trajectory and percentage change (per year) in fat mass at age specified (95% CI)	Mean difference (95% CI) per SD higher child GRS	Mean difference (95% CI) per SD higher adult GRS
9	7.2 (7.1, 7.4) kg	10.5 (8.6,12.4) %	5.1 (3.2,6.9) %
9–13	18.1 (17.3, 18.9) %/y	−0.1 (-0.5, 0.3) %/y	0.2 (-0.2, 0.6) %/y
13	14.1 (13.8, 14.4) kg	10.0 (8.0,12.0) %	5.9 (4.0,7.9) %
13–15	10.2 (9.4, 11.1) %/y	−1.1 (-1.9, -0.4) %/y	−0.9 (-1.7, -0.2) %/y
15	17.1 (16.8, 17.4) kg	7.5 (5.4,9.7) %	4.0 (1.9,6.1) %
15–18	6.4 (5.9, 6.8) %/y	0.01 (-0.5, 0.5) %/y	0.2 (-0.3, 0.6) %/y
18	20.6 (20.2, 21.0) kg	7.5 (5.2,9.8) %	4.4 (2.2,6.7) %
18–25	2.2 (2.0, 2.5) %/y	−0.3 (-0.6, -0.1) %/y	0.2 (-0.1, 0.4) %/y
25	24.0 (23.5, 24.6) kg	5.0 (2.5,7.5) %	5.8 (3.3,8.3) %

Mean trajectory is centred on the sex-specific mean of the child [female mean: 0.0086 (SD: 0.0005)] and adult [female mean: 0.0079 (SD: 0.0004)] GRS. The difference in fat mass per SD higher child or adult GRS is back-transformed from the log scale for ease of interpretation and is a ratio of geometric means, expressed as a percentage difference. GRS, genetic risk score; SD, standard deviation; CI, confidence interval; y, years.

Unadjusted findings (from models that did not adjust for the other GRS) were broadly similar to adjusted findings among males and females (Supplementary Tables S2 and S3, available as Supplementary data at *IJE* online). Mutually adjusted models using sex-combined GRS were also used. In males, a greater effect of the childhood GRS was observed in these models at earlier ages. For example, at age 9 y a 1-SD higher childhood GRS was associated with 14.9% (95% CI 12.4, 17.5) higher fat mass (see Supplementary Table S4, available as Supplementary data at *IJE* online) compared with the 8.8% (95% CI 6.3, 11.3) difference seen in the sex-specific GRS model. In females, these models produced similar findings to our main models, albeit with slightly higher point estimates (Supplementary Table S5, available as Supplementary data at *IJE* online). Associations for childhood and adulthood GRS scores with trunk and peripheral fat mass were similar to our main findings on total fat mass (Supplementary Figure S2, available as Supplementary data at *IJE* online).

### Estimated effects of childhood and adulthood body size GRSs on trajectories of total lean mass

Supplementary Figure S1C and D (available as Supplementary data at *IJE* online) shows the mean sex-specific trajectories of total lean mass from 9 to 25 y for: (i) participants with the mean childhood GRS and mean adulthood GRS; (ii) participants with a 1-SD higher childhood GRS compared with the mean; and (iii) participants with a 1-SD higher adulthood GRS compared with the mean. Compared with a male with mean childhood and adulthood GRS, a 1-SD higher childhood and adulthood GRS was associated with small differences in total lean mass at age 9 y: 0.9% (95% CI 0.4, 1.3) and 0.5% (95% CI 0.03, 0.9) per SD higher childhood and adulthood GRS, respectively (Supplementary Table S6, available as Supplementary data at *IJE* online; and Figure 1C). The childhood GRS was associated with small differences in rates of change in total lean mass from 9 to 25 y which differed in direction across spline periods, whereas the adulthood GRS was associated with similar rates of change in total lean mass from 9 to 25 y. By age 25 y, a 1-SD higher childhood and adulthood GRS was associated with 1.1% (95% CI 0.3, 2.0) and 1.4% (95% CI 0.5, 2.3) higher total lean mass, respectively, among males. Findings were broadly similar for females (Supplementary Table S7, available as Supplementary data at *IJE* online; and Figure 1D). Unadjusted findings (from models that did not adjust for the other GRS score) and results of sex-combined GRS applied to sex-specific samples were broadly similar to adjusted findings, see Supplementary Tables S8 and S9 for unadjusted results and Supplementary Tables S10 and S11 for mutually adjusted results using the sex-combined GRS (available as Supplementary data at *IJE* online).

### Sex-combined analyses of body size GRS effects on total fat and total lean mass

As shown in Supplementary Figure S1, trajectories of fat and lean mass are sex specific and therefore all prior analyses are sex specific. For comparison and completeness, we also examined associations of the sex-combined GRSs with body composition outcomes applied to a sex-combined sample. Supplementary Figure S3 (available as Supplementary data at *IJE* online) shows the associations of a 1-SD increase of the childhood and adulthood body size GRS with total fat and total lean mass from the ages of 9 to 25. For these models we see that a + 1-SD increase in childhood GRS is associated with a greater increase in total fat mass than a + 1-SD increase in adulthood GRS, between the ages of 9 and 15 (per 1-SD childhood GRS at age 13: 12.7% (95% CI 10.9, 14.5) vs per 1-SD adulthood GRS at age 13: 6.7% (95% CI 5.0, 8.3)), before attenuating at age 18 and beginning to reverse at age 25. Associations with differences in total lean mass were much smaller than for total fat mass, as seen previously. These results are available in Supplementary Tables S12 and S13 (available as Supplementary data at *IJE* online).

We analysed the percentage of variance in total fat mass and total lean mass which was explained by the GRS for females and males. In all instances, the sex-combined scores explained a greater amount of variance than their sex-specific counterparts. These results are available in Supplementary Tables S14–S17 (available as Supplementary data at *IJE* online).

### Single SNP effects on total fat mass and total lean mass

We also ran sex-specific analyses using single SNPs (from the sex-specific GRSs) to determine the per one allele raised SNP effects on trajectories of total fat mass and total lean mass in males and females at each time point. At each time point there were numerous associations found between SNPs and fat mass outcomes for males and females; this is also true for the total lean mass outcomes, albeit with much smaller effect sizes than for fat mass (as seen in the GRS models). The full results of these analyses are available in Supplementary File 4 (available as Supplementary data at *IJE* online).

## Discussion

In this prospective cohort study, we aimed to estimate the effects of life stage-specific genetic susceptibility to higher body size on trajectories of objectively measured body fat and lean mass from childhood to young adulthood. Our findings show that genetic variants for body size are more strongly associated with adiposity than lean mass from childhood to early adulthood. They demonstrate that sex-specific childhood variants are more strongly associated with adiposity in females than adulthood variants until early adulthood, whereas childhood and adulthood variants are similarly associated with adiposity across early life in males. We also show that the sex-combined childhood GRS has stronger effects in childhood for both males and females, and as such it would be sensible for future analyses to prioritize sex-combined GRSs, including for sex-specific analyses. Our findings may inform future studies that use genetics to investigate the causes and impact of adiposity at different life stages.

Recently, a number of GWAS have been run in the Norwegian Mother, Father and Child Cohort Study (MoBa) between birth and 8 years of age, identifying novel SNPs that are associated with BMI specifically at different time points throughout this age range, suggesting that the genetic drivers of BMI change throughout early childhood.^28^ Khera *et al*. previously derived a genome-wide polygenic score (GPS) for BMI, using adult BMI-linked SNPs and combining a large set of SNPs with small effect sizes, with the aim of prediction (not causation).^29^ In that study, repeated measures of weight from ALSPAC, recorded from birth to 18 years of age, were used to establish that this GPS for adult BMI is associated with increased body weight already in early childhood, with associations further strengthening into young adulthood. However, little is known about whether distinct genetic influences exist on childhood vs adulthood adiposity, within a causal inference framework.

In our present study, we used a smaller set of SNPs that were more strongly associated (*P* <5 x 10^-8^) with generally measured ‘body size’, to estimate their effects on more objective measures of body fat and lean mass at different stages of the early life course. This was important because the source GWAS for these SNPs, although substantially larger than other GWAS of childhood BMI, used a self-reported measure of recalled body size relative to peers when aged 10 years (assessed via questionnaire several decades later). Despite these variants having been used in several life course MR studies to instrument ‘body size’ in childhood (with the assumption that this indicates childhood adiposity), it is unknown whether these variants are truly instrumenting adiposity as opposed to lean mass, how these variants affect trajectories of fat and lean mass throughout childhood and whether these effects differ by sex. Our results suggest that genetic variants for body size are more strongly associated with body fat than lean mass across early life in both sexes. Moreover, our findings show that in females during childhood and adolescence, the childhood body size SNPs are more strongly associated with adiposity than the adulthood SNPs, whereas childhood and adulthood SNPs are similarly associated with adiposity in males from childhood to early adulthood. We note that when using the combined-sex SNPs, the males display results more similar to females (and female effect estimates increase), suggesting the sex-combined SNPs are likely a better instrument for adiposity than the sex-specific SNPs, perhaps in part due to increased statistical power due to a larger number of constituent SNPs in the sex-combined GRS.

We also analysed the effects of single SNPs on total body fat and lean mass. We observe that the SNPs typically have larger (albeit less precise) effects on total fat mass than on lean mass, as would be expected in line with our GRS results. We do note however, that a small number of SNPs appear to have negative effects on total fat mass; these findings differ from those of Richardson *et al.* which showed the same SNPs as increasing total fat mass when examined in the UK Biobank dataset [identified by measured adult BMI (mean age 56.6)]. This is particularly interesting for the adult female GWAS identified rs12364470 SNP; we note that by age 25 the effect is null (albeit directionally consistent), which brings into question if the G allele reduces fat mass in early life (as our results suggest) before the T allele becomes associated with reduced fat mass in later life (as per previous literature^11^). We note that power is low using single SNP effects and in a much smaller sample (ALSPAC: ∼7000 individuals, UK Biobank ∼450 000 individuals), in comparison with GRS, and as such this finding may be a false positive.

We speculate that a potential biological explanation for these findings may be due to childhood identified SNPs being more strongly linked to ‘biological’ (e.g. appetite, metabolism) propensity to higher adiposity, whereas adulthood SNPs may have stronger associations with social/behavioural differences (e.g. greater freedom in feeding self, education, risk-taking behaviours). It is also clear that larger body size in children is associated with earlier onset of puberty in boys and girls.^30^^,^^31^ In a post-hoc analysis to interrogate the biology of our findings, we took the two SNPs with the strongest effect estimates (with a 95% CI that did not cross the null) from each GRS (for fat mass) and identified their strongest associations with traits in the literature, using PhenoScanner.^32^^,^^33^ In females, the top two SNPs were rs62106258 and rs41279738 for both the child and adult GRS; rs62106258 is most strongly associated with basal metabolic rate (BMR), adult BMI, and age at menarche, whereas rs41279738 (situated in the GPR61 gene) is also most strongly associated with BMR and adult BMI. In males, the top two child GRS SNPs were rs77165542, associated with BMR, adult BMI and self-reported hypothyroidism, and the rs61978655 SNP (situated in the PRKD1 gene) which is associated with BMR and adult BMI. From the male adult GRS, the top two SNPs were rs7240682, associated with BMR, adult BMI and type 2 diabetes, and the rs62048402 SNP [situated in the eponymous fat mass- and obesity-associated protein (FTO) gene] which is associated with a range of phenotypes such as BMR, BMI, type 2 diabetes and relative age at emergence of facial hair.

As we demonstrate, the genetic variants which are used in our analyses clearly have much stronger relative associations with fat mass than lean mass. How well these findings in particular would transfer to prevention/intervention measures is unclear. However, we believe furthering our understanding of genetic predisposition to higher fat mass may aid in reduction of medical stigma (assuming clinical education around this subject); for example, it has been shown that health care professionals exhibit weight bias against larger patients,^34^ although it is unclear if this has downstream effects on patient care and clinical outcomes. Extending our study in the future to later adulthood would further improve understanding of whether genetic risk of childhood adiposity influences body composition in later life, and precisely when the genetic variants for adulthood body size establish themselves as more greatly influential for adiposity.

The limitations of this study include the restriction of analyses to participants identified by parents as being of a White ethnicity (given the European sample from which genetic variants were discovered) and therefore generalizability of our findings to other groups.^35^ The DXA measures used to quantify fat and lean mass are considered superior to highly indirect measures such as BMI or waist circumference, but DXA measures are less precise than MRI,^36^^,^^37^ and unable to measure ectopic fat and may not have uniform precision across the range of BMI values.^38^ ALSPAC data are longitudinal and there are potential issues arising from loss to follow-up over time which are unlikely to be at random.^39^ This may bias results, and we note that individuals included in our analysis are from higher socioeconomic backgrounds than those not included.

## Conclusions

Genetic variants for body size are more strongly associated with adiposity than lean mass from childhood to early adulthood. Based on sex-combined GRSs (which explain a greater percentage of outcome variance than sex-specific GRSs), childhood variants are more strongly associated with adiposity in females and males until early adulthood. These findings help to reveal the genetic basis for adiposity across the life course and may inform instrument selection for future studies that use genetics to investigate the causes and impact of adiposity at different life stages.

## Ethics approval

The study has been described elsewhere in detail and ethical approval for the study was obtained from the ALSPAC Ethics and Law Committee and the local research ethics committees.

## Supplementary Material

dyad029_Supplementary_Data

## Data Availability

Individual-level ALSPAC data are available following an application. This process of managed access is detailed at [www.bristol.ac.uk/alspac/researchers/access]. Cohort details and data descriptions for ALSPAC are publicly available at the same web address.
