# Supplementary material for: Life course effects of genetic susceptibility to higher body size on body fat and lean mass: prospective cohort study
Source: Int J Epidemiol. 2023 Mar 23;52(5):1377–87. doi: 10.1093/ije/dyad029 (PMC10555894; doi:10.1093/ije/dyad029)
Supplement: dyad029_Supplementary_Data [file dyad029_supplementary_data.zip › dyad029_Supplementary_Data/ije-2022-06-0757-File006.docx]

**Model Outlines**

Primary models are sex stratified and use sex-specific GRS values (with and without mutual adjustment), secondary models are also sex stratified and use sex-combined GRS values (with and without mutual adjustment), and the tertiary models are sex-combined and use sex-combined GRS with mutual adjustment.

**Primary/Secondary/Tertiary model with mutual adjustment**

$$DXA Outcome \sim1+Spline1+Spline2+Spline3+Spline4+Height+GRS_{Child}+GRS_{Adult}+Spline1*GRS_{Child}+ Spline1*GRS_{Adult}+Spline2*GRS_{Child}+Spline2*GRS_{Adult}+ Spline3*GRS_{Child}+ Spline3*GRS_{Adult}+ Spline4*GRS_{Child}+Spline4*GRS_{Adult}+\left( 1 | Spline1+Spline2+Spline3+Spline4 \right)+(1|ID)$$

**Primary/Secondary model without mutual adjustment**

$$DXA Outcome \sim1+Spline1+Spline2+Spline3+Spline4+Height+GRS_{Child/Adult}+Spline1*GRS_{Child/Adult}+ Spline2*GRS_{Child/Adult}+ Spline3*GRS_{Child/Adult}+ Spline4*GRS_{Child/Adult}+\left( 1 | Spline1+Spline2+Spline3+Spline4 \right)+(1|ID)$$

**Single SNP model**

$$DXA Outcome \sim1+Spline1+Spline2+Spline3+Spline4+Height+SNP+Spline1*SNP+ Spline2*SNP+ Spline3*SNP+ Spline4*SNP+\left( 1 | Spline1+Spline2+Spline3+Spline4 \right)+(1|ID)$$
